# Advancing AAV technology—From promise to product

**DOI:** 10.1016/j.omta.2026.201672

**Published:** 2026-01-20

**Authors:** Juliette Hordeaux, Allison M. Keeler, Daniel Stone

**Affiliations:** 1 Gemma Biotherapeutics, Inc., Philadelphia, PA, USA; 2 Horae Gene Therapy Center, Department of Genetic and Cellular Medicine, NeroNexus Institute, Li Weibo Institute for Rare Diseases Research, University of Massachusetts Chan Medical School, Worcester, MA, USA; 3 Vaccine and Infectious Disease Division, Fred Hutchinson Cancer Center, Seattle, WA, USA

## Main text

*Molecular Therapy - Methods and Clinical Development* is delighted to present a second collection of AAV-focused papers. This collection, curated by members of our scientific editorial team with AAV expertise, features translational, and preclinical studies published from 2024–25 involving novel AAV-based therapeutics. We have included studies that encompass AAV safety and immunogenicity; AAV-based gene editing and *ex vivo* therapeutics; AAV-based therapy evaluation in small animal models of disease; as well as AAV-based therapy evaluation in large animals and humans.

### AAV safety—Redosing and host immune responses

Host immune responses are significant hurdles to the clinical use of AAV-based gene therapeutics as antibody and T cell responses developed against the capsid and/or transgene can inhibit target cell transduction, and limit persistence of transgene expression. This section introduces studies that investigate all aspects of AAV safety, with a specific focus on studies that address the issues of vector redosing and host immune responses against AAV-based therapies. The ability to administer multiple doses of AAV in the clinical setting is hindered by the development of neutralizing antibodies (NAbs) against the capsid that prevent cellular entry and subsequent gene delivery. Two studies in this section demonstrate that redosing of AAV can be achieved with approaches that utilize either novel isolates which can evade NAbs mounted against highly similar capsids, or use lipid nanoparticles to shield AAV vectors from host NAbs.^1^^,^^2^ Another featured study in this section investigated host T cell responses to the exogenous bacterial *Sa*Cas9 endonuclease when delivered via an AAV vector. They identified a known T cell epitope in SaCas9 that triggered strong cytotoxic T cell activity and target cell killing, and engineered Cas9 variants with modified immunogenic epitopes, successfully reducing immune activation.^3^

### Gene editing and *ex vivo* applications

AAV vectors have become an essential component of the gene editing toolbox as they are being used as homology directed repair (HDR) donors to create precise gene edits at specific chromosomal loci across a wide range of cell types and tissues. They are also being extensively used to deliver exogenous gene-editing enzymes, such as targeted endonucleases, nickases, base editors, or prime editors into desired target cell populations. Among the papers included in this section is a study that improves the efficiency of AAV-mediated HDR in hematopoietic stem and progenitor cells (HSPC) *ex vivo* by inhibiting the PKc pathway and biasing double-strand break repair from non-homologous end joining (NHEJ) toward HDR.^4^ This section also includes a detailed investigation of off-target cleavage events *in vivo* (that can potentially lead to unwanted mutations or AAV vector insertions) following co-administration of one AAV vector expressing Cas9 and another AAV expressing a sgRNA that targets the mouse factor IX chromosomal locus.^5^

### AAV application to disease—Small animal studies

According to the Online Mendelian inheritance in Man (OMIM) database over 5000 human genes carry phenotype-causing mutations. There are also thousands of human pathogens, hundreds of different cancers, and numerous other acquired diseases. Thus, there are many diseases and disorders that are potentially amenable to cell and gene therapies. In this section we introduce studies targeting a specific disease with an AAV vector, focusing on small animal models of disease. Highlighted examples include proof of concept studies for AAV-based gene replacement therapies targeting the monogenic disorders mucopolysaccharidosis type I (MPS I), a rare genetic disease caused by deficiency in α-L-iduronidase (IDUA), and Crigler-Najjar syndrome, a rare genetic liver disease caused by mutations in the *UGT1A1* gene.^6^^,^^7^ Both studies used knockout mouse or rat models to demonstrate therapeutic efficiency following enzyme replacement in a model that recapitulates the disease genotype and/or phenotype.

### AAV application to disease—Large animal and human studies

Large animal models are indispensable in translation from preclinical proof-of-concept to human studies, serving distinct but complementary roles in safety and efficacy evaluation. Healthy large animals, particularly nonhuman primates (NHPs), present more comparable immune responses, organ size, and biodistribution patterns that allow for more predictive assessments of dose limiting toxicity, off-target effects, and vector pharmacokinetics and pharmacodynamics than rodent models. In addition, naturally occurring genetic disease models in animals, such as dogs, cats, and sheep are essential for demonstrating the scalability of gene therapy approaches.^8^ This section highlights preclinical studies that elegantly and ethically use large animal models to lay the groundwork that will de-risk clinical trials. It also highlights early human studies related to the development of AAV gene therapies. One featured study investigated gene transfer efficacy and safety in fetal lambs that received AAV9 vectors via the umbilical vein or fetuses that received AAV9 through the cisterna magna,^9^ expanding on previous work from their group in the field of prenatal somatic gene therapy for monogenic disorders. Another featured study reports the prevalence of neutralizing antibodies against 6 clinically relevant isolates (AAV1, AAV5, AAV6, AAV8, AAV9, and AAVrh74) within a large cohort of donors from 10 countries.^10^

### Summary

We hope this second AAV-focused collection demonstrates how AAV vectors are being used and evaluated for the treatment of inherited and acquired diseases. There is still much to learn about AAV and, more importantly, host responses associated with its administration, particularly considering serious adverse events and deaths in patients receiving high systemic doses. Nevertheless, optimism remains that safe AAV-based therapies can still be developed for clinical use against a broad array of diseases. As this field evolves, *MTMCD* continues to publish studies describing AAV-based therapeutics and their transition from concept to pharmaceutical. We hope you find our AAV-themed special collections informative and useful.
